# Improvements in Blood Profiles of Canines Naturally Infected with Triple Blood Pathogens (*Babesia vogeli*, *Ehrlichia canis*, and *Anaplasma platys*) Subsequent to Doxycycline Monotherapy

**DOI:** 10.3390/ani14243714

**Published:** 2024-12-23

**Authors:** Tuempong Wongtawan, Narin Sontigun, Kanpapat Boonchuay, Phatcharaporn Chiawwit, Oraphan Wongtawan, Orachun Hayakijkosol, Worakan Boonhoh

**Affiliations:** 1 Akkhraratchakumari Veterinary College, Walailak University, Nakhon Si Thammarat 80160, Thailand; tuempong.wo@mail.wu.ac.th (T.W.); kanpapat.bo@mail.wu.ac.th (K.B.); phatcharaporn.cw@gmail.com (P.C.); oraphan.kanth@gmail.com (O.W.); 2 One Health Research Centre, Walailak University, Nakhon Si Thammarat 80160, Thailand; 3 Center of Excellence Research for Melioidosis and Other Microorganism, Walailak University, Nakhon Si Thammarat 80160, Thailand; 4 Office of Administrative Interdisciplinary Program on Agricultural Technology, School of Agricultural Technology, King Mongkut’s Institute of Technology Ladkrabang, Bangkok 10520, Thailand; narin.so@kmitl.ac.th; 5 Division of Tropical Health and Medicine, College of Public Health, Medical and Veterinary Sciences, James Cook University, Brisbane City, QLD 4000, Australia; orachun.hayakijkosol1@jcu.edu.au; 6 Center of Excellence in Innovation on Essential Oil and Bioactive Compounds, Walailak University, Nakhon Si Thammarat 80160, Thailand

**Keywords:** blood parasite, dogs, hematology, multiple blood pathogen infection, tick-borne pathogens

## Abstract

Multiple blood pathogen infections are increasingly found in many areas, particularly in tropical regions. This study aimed to investigate the effectiveness and safety of using doxycycline monotherapy to treat triple blood pathogen infection in sheltered dogs. A total of 375 sheltered dogs were screened for blood pathogen using polymerase chain reaction technique (PCR). There were 34 dogs with triple infection (*Babesia vogeli*, *Ehrlichia canis*, and *Anaplasma platys*), and most dogs displayed anemia and thrombocytopenia. These dogs were treated with doxycycline (10 mg/kg/day) orally for four weeks, and their blood profiles were monitored. Almost all pathogens were undetectable by PCR by day 14 and pathogens were all cleared by day 28. Most blood profiles significantly improved after 14 days. The improvement continued after 28 days. The red blood cell and platelet count (PLT) were increased in similar trends between mild-to-moderate and severe thrombocytopenia groups. The average PLT in the mild-to-moderate thrombocytopenia group raised to normal level by day 14, whereas in the severe group, it was gradually increased to normal level by day 70. We propose using doxycycline monotherapy for 28 days to eliminate all pathogens, and facilitate recovery and welfare in dogs suffering with triple blood pathogen infections.

## Simple Summary

The presence of multiple types of blood pathogens often results in more severe symptoms and threatens dogs’ health and welfare more than a single blood pathogen infection. However, the standard protocol for treating multiple blood pathogen infection in dogs is still unavailable. The present study suggests that triple blood pathogen infection with *Babesia canis vogeli*, *Ehrlichia canis*, and *Anaplasma platys* can cause severe anemia and thrombocytopenia. Applying doxycycline as a monotherapy at a dosage of 10 mg/kg/day orally once daily for 28 days effectively eliminates all pathogens and promotes recovery in dogs naturally co-infected with *B. vogali*, *E. canis*, and *A. platys.*

## 1. Introduction

The transmission of tick-borne diseases is escalating throughout tropical regions, such as Southeast Asia and Thailand, causing significant risks to animal health [1,2,3]. In 2023, the estimated dog population that was registered in Thailand was approximately three million, with the majority being owned pets and the remaining being free-roaming and stray dogs, in which the current population figures are not available in these groups but increasing every year [4]. In Thailand, there are about 30 dog shelters, and each shelter typically houses 500 dogs. These shelters have turned into vector-borne pathogen hubs because of the difficulties in maintaining the health of the large dog population [2,3]. These dogs are susceptible to multiple pathogens infection and the most common blood pathogens in dogs in Thailand are *Ehrlichia canis*, followed by *Anaplasma platys* and *Babesia canis vogeli* [3,5,6,7]. These blood pathogens are easily and rapidly transmitted via a common tick species in Thailand and other tropical regions, *Rhipicephalus sanguineus* or the brown dog tick [2,3]. Clinical signs and symptoms of blood-borne pathogen infection in dogs are fever, decreased appetite, pale mucous membranes, cachexia, weight reduction, and lymphadenopathy [3]. Infections with multiple blood pathogens can lead to more severe anemia and thrombocytopenia than single blood parasite infection [3,6,7]. Most of the sheltered dogs with vector-borne pathogen infection exhibit subclinical conditions unless they are severely infected with high levels of parasitemia [2,3].

There are several methods to assess the blood pathogen infections in dogs, including commercial test kits, microscopic examination, and polymerase chain reaction (PCR). Among all these procedures, the polymerase chain reaction-based techniques are the most specific and sensitive, particularly *E. canis* and *B. vogeli* [2,3,6].

The recommended treatment for ehrlichiosis and anaplasmosis is doxycycline [8]. For both conditions, doxycycline is advised for a minimum duration of 28 days [9,10]. Common side effects of doxycycline include hepatic injury [11]. However, doxycycline is not typically the first-choice medicine to treat canine babesiosis. The recommended medications for treating babesiosis and hepatozoonosis in dogs are imidocarb diproprionate (intramuscular injection) or a combination of atovaquone and azithromycin (oral) [12,13]. Common side effects of imidocarb diproprionate include vomiting and hypersalivation [14,15], while the combination of atovaquone and azithromycin can cause gastrointestinal irritation, such as vomiting, diarrhea, and rash [16].

However, when multiple blood pathogen infections are present, a single medication like doxycycline is often chosen for convenience to manage the infections in sheltered dogs and minimize side effects of other combination medicines. Doxycycline was used to treat experimental double infection of *E. canis* and *A. platys* for 28 days [17] and naturally concurrent infections of canine ehrlichiosis and babesiosis for two weeks [18], resulting in improved hematology profile and the pathogens becoming undetectable.

Currently, there is no established treatment protocol for triple blood pathogen infections involving babesiosis, ehrlichiosis, and anaplasmosis. The objective of this study was to evaluate the effectiveness and safety of doxycycline monotherapy in treating dogs naturally infected with triple blood pathogens. This could be a useful tool for improving the health and welfare of sheltered, free-roaming, stray, or even pet dogs that are naturally infected with multiple blood pathogens.

## 2. Materials and Methods

### 2.1. Ethical Statement

This study was approved by the Institutional Animal Care and Use Committee of Walailak University (WU-ACUC-66041) and the Walailak University Biosafety Committee (WU-IBC-66-047).

### 2.2. Animals and Blood Samples

There were 375 mixed breed dogs in a dog shelter in Nakhon Si Thammarat, Thailand. They ranged in age, approximately 2–7 years, and body weight ranged from 7.5 to 20 kg on the day of blood collection. The dogs were collected blood 3 mL via cephalic or saphenous veins by a veterinarian, and transferred to an ethylenediaminetetraacetic acid (EDTA) blood tube for 2 mL and a clot activator blood tube for 1 mL to check for blood profile, blood pathogens, and general health. The dogs were restrained by a veterinary technician and dog keepers from the dog shelter. All the dogs were vaccinated with Biocan Novel DHPPi/L4R (Interpharma, Bangkok, Thailand) subcutaneously; the combination vaccine included distemper, adenovirus type 2, parainfluenza, parvovirus, leptospira, coronavirus, and rabies and they were regularly injected with ivermectin subcutaneously at a dosage of 400 mcg/kg to treat and prevent ecto- and endoparasites.

### 2.3. Hematology and Serum Biochemistry Analysis

Hematology and serum biochemistry were performed using an automatic Procyte Dx machine (IDEXX Laboratories, Westbrook, ME, USA). The hematology parameters measured included red blood cell count (RBC), hemoglobin concentration (HGB), hematocrit (HCT), reticulocyte counts (REC), platelet count (PLT), mean platelet volume (MPV), total white blood cell count (WBC), neutrophil count (NEU), lymphocyte count (LYM), eosinophil count (EOS), monocyte count (MON), and platelet count (PLT). The serum biochemistry analysis included blood urea nitrogen (BUN), creatinine (CRE), aspartate aminotransferase (AST), alanine aminotransferase (ALT), alkaline phosphatase (ALP), and total protein (TP). Reference values for hematology and serum biochemistry were based on previous studies of healthy dogs in southern Thailand [3,5].

### 2.4. Treatment of Multiple Pathogen Infection

There were 34 dogs from the dog shelter that infected with triple blood pathogens (*B. vogeli*, *E. canis*, and *A. platys*) and they were isolated in an isolation zone of the dog shelter and taken to a small animal teaching hospital, Akkhraratchakumari veterinary college, for examination every two weeks. All of the infected dogs (N = 161), presenting with either single, double, or triple infections, were prescribed doxycycline monotherapy at a dosage of 10 mg/kg/day orally once daily for four weeks. For supplementary treatment, dogs with anemia were given Dr.Choice-Ferro-BTM (Interpharma, Bangkok, Thailand), which contains ferrous amino acid chelate, copper amino acid chelate, multivitamins, and folic acid. Dogs showing symptoms of liver injury were provided with Dr.Choice-Livo-BTM (Interpharma, Bangkok, Thailand), which includes silymarin, glutathione, folic acid, choline, inositol, amino acids, and multivitamins. Dogs that displayed symptoms of kidney injury were treated with fluid therapy (acetate ringer’s solution via intravenous and subcutaneous routes).

Hematology and serum biochemistry of the triple pathogen-infected dogs were monitored before treatment (day 0) and after treatment with doxycycline every two weeks (at day 14, day 28, day 42, day 56, and day 70). After finishing the blood collection at day 70, the dogs were moved back to their previous shelter zones.

### 2.5. Detecting Blood Pathogen with Polymerase Chain Reaction (PCR)

The EDTA blood samples were kept in an –80 °C freezer until blood pathogens were analyzed. DNA from whole blood was extracted using the gSYNCTM DNA Extraction Kit (Tissue/blood) (Geneaid, New Taipei City, Taiwan), following the manufacturer’s instructions. DNA concentration was measured using a NanoDrop™ spectrophotometer (ThermoFisher Scientific, Waltham, MA, USA).

PCR primers (Table 1) were utilized according to previous studies [19,20,21,22,23]. The PCR reaction mix contained 6.25 µL of DreamTaq Green Master Mix (2×) (Thermo Scientific, Vilnius, Lithuania), 1–2 µL of DNA template (100–200 ng/µL), 0.5 µL of primer (0.4 µM), and nuclease-free water to a final volume of 12.5 µL. The reactions were conducted using the Mastercycler Pro S machine (Eppendorf AG, Hamburg, Germany). The cycling conditions were as follows: an initial denaturation step at 95 °C for 3 min, followed by 35 cycles of denaturation at 95 °C for 30 s, annealing at 52–58 °C (depending on the pathogen, see Table 1) for 30 s, and extension at 72 °C for one minute. A final extension step at 72 °C for five minutes was also included.

The positive control consisted of DNA from known blood parasites collected in previous studies [2], while the negative control was nuclease-free water. PCR products were visualized on a 1.5% agarose gel in 1× Tris acetate EDTA buffer, stained with SERVA DNA Stain G (SERVA, Heidelberg, Germany), and observed under UV light using the ChemiDoc™ Imaging System (Bio-Rad, Hercules, CA, USA).

### 2.6. Statistical Analysis

Jamovi 2.3.9 software (accessed on 7 May 2024; https://www.jamovi.org/) was used for statistical analysis. Hematology and serum biochemistry data for the dogs were represented as mean ± standard deviation (SD). Statistical differences in hematology and serum biochemistry profiles among treatment days were compared using a repeated measures ANOVA followed by a post-hoc Tukey test.

The prevalence of hematology abnormalities (such as anemia and thrombocytopenia) and serum biochemistry abnormalities (e.g., elevated BUN) was expressed as percentages. Statistical significance among treatment groups was analyzed using the chi-square test. A *p*-value of less than 0.05 was considered statistically significant, and a *p*-value of less than 0.01 was considered highly statistically significant.

## 3. Results

### 3.1. Screening Blood Pathogens

Out of all of the 375 sheltered dogs, 57.07% (N = 214) were negative for blood pathogens, while 42.93% (N = 161) were positive. Among the infected dogs (N = 161), 54.04% (N = 87) had a single infection, 24.84% (N = 40) had a double infection, and 21.12% (N = 34) had a triple infection. The details of single, double, and triple pathogen infections are shown in Table 2. Triple infections involved *E. canis*, *A. platys*, and *B. vogeli*.

Specifically, *E. canis* was the most common pathogen, found in 95.03% (N = 153) of infected dogs, with a prevalence of 40.80% among all dogs in the shelter. The prevalence of each pathogen is shown in Table 2. Notably, *Trypanosome* spp. was detected in one dog.

### 3.2. The Presence of Blood Pathogens After Treatment with Doxycycline

**Figure 1:**
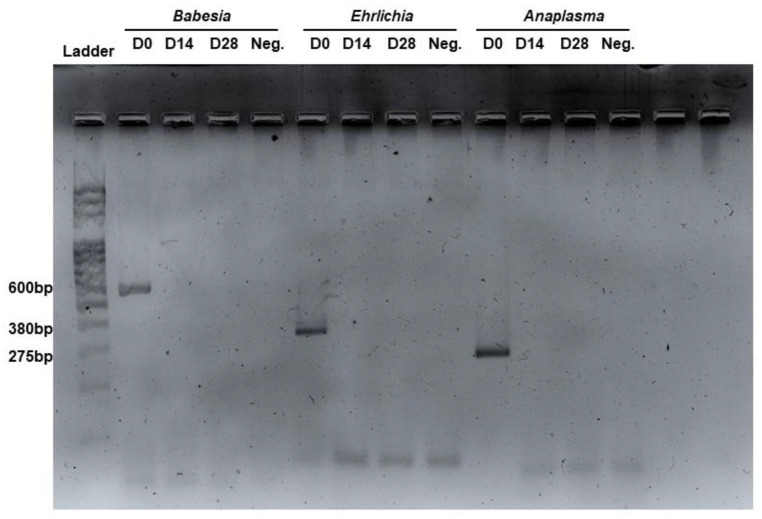
Gel electrophoresis of polymerase chain reaction (PCR) from blood samples of the dogs at Day 0, 14, and 28.

No pathogens were detected after 14 days of treatment in cases of single and double pathogen infections. However, in cases of triple pathogen infections, almost none of the pathogens were detected after 14 days (Figure 1), with the exception of one dog that still tested positive for *B. vogeli*, which became undetectable after 28 days.

### 3.3. Red Blood Cell and Platelet Parameters of Triple Blood Pathogen Infection Before and After Treatment

Generally, the number of erythrocytes, HCT, HGB, and PLT showed a continuous increase (Table 3), while reticulocyte tended to decrease from day 0 to day 70 (Table 3). Statistically, RBCs significantly increased from day 0 to day 42 (*p* < 0.01), but there was no significant difference from day 42 to day 70 (*p* > 0.05). HCT significantly increased from day 0 to day 42 (*p* < 0.05), but no significant difference was observed from day 42 to day 70 (*p* > 0.05). HGB significantly increased from day 0 to day 42 (*p* < 0.05), but there was no significant change after that. Additionally, there was no significant difference in RET (*p* > 0.05). PLT significantly increased (*p* < 0.01) from day 0 to day 70, while MPV significantly reduced from day 0 to day 14 (*p* < 0.01) (Table 3).

### 3.4. Prevalence of Anemia and Thrombocytopenia Before and After Treatment

The number and percentage of dogs with anemia and thrombocytopenia are shown in Table 4. Anemia, which is characterized by reduced levels of RBC, HCT, and/or HGB, was observed to range from 67.65% (low RBC) to 88.24% (low HCT or HGB) in the multiple-blood-pathogen-infected dogs. A statistically significant reduction in anemia prevalence was noted starting from day 14 post-treatment (*p* < 0.02). This decline continued progressively from day 14 to day 70, with the lowest prevalence recorded at 11.76% (RBC) and 35.29% (HCT and HGB) (Table 4).

Thrombocytopenia was initially prevalent in 91.18% of the subjects prior to treatment. A statistically significant decrease in thrombocytopenia was observed post-treatment (*p* < 0.01). The prevalence of thrombocytopenia consistently declined from day 14 to day 70, reaching a minimum prevalence of 32.35% (Table 4).

### 3.5. White Blood Cell Parameters of Triple Pathogen Infection Before and After Treatment

The change in white blood cells is presented in Table 5. There was a slight decrease in WBC, but it was not significantly changed (*p* > 0.05). Specifically, only the number of MON and EOS showed statistically significant changes. The number of monocytes significantly decreased after treatment from day 0 to day 42 (*p* < 0.01), with no significant difference from day 42 to day 70 (*p* > 0.05). Conversely, the EOS significantly increased (*p* < 0.01) from day 0 to day 42 and then remained stable (*p* > 0.05) (Table 5).

### 3.6. Serum Biochemistry Before and After Treatment

BUN, AST, ALT, and TP levels were generally higher before treatment and decreased afterward (Table 6). However, there was no significant difference in BUN, AST, CRE, and TP (*p* > 0.05). AST significantly decreased from day 0 to day 14 (*p* < 0.01), with no further statistical changes observed (*p* > 0.05). ALP showed a significant reduction between day 0 and day 70 (*p* < 0.05) (Table 6).

### 3.7. The Prevalence of Hepatic and Renal Injuries

The prevalence of hepatic and renal injuries before and after treatment is shown in Table 7. For parameters associated with renal function, the highest prevalence was seen in elevated BUN, at 20.69% (N = 7) before treatment, which reduced to 0% by day 42. The prevalence of elevated CRE was 8.82% before treatment, decreasing to 2.94% from day 42 to day 70.

Regarding liver-function-associated enzymes, the occurrence of elevated ALP was 11.76% (N = 4) before treatment, reducing to 0% by day 28. The number of dogs with elevated AST was 5.88% (N = 2) on day 0, disappearing after day 14. An elevated ALT was found in 2.94% (N = 1) of dogs at day 0 to 42 and increasing to 5.88% (N = 2) at days 56 to 70 (Table 7).

For renal injury parameters, an elevated CRE was found in 8.82% of dogs (N = 3) initially, reducing to 2.94% by day 42 and remaining at that level until day 70. An elevated BUN was found in 20.69% of dogs (N = 7) before treatment, reducing to 0% by day 42. However, there was no statistically significant difference (*p* > 0.05) in the prevalence of hepatic and renal injury before and after treatment (Table 7).

### 3.8. Trend of Recovery

**Figure 2:**
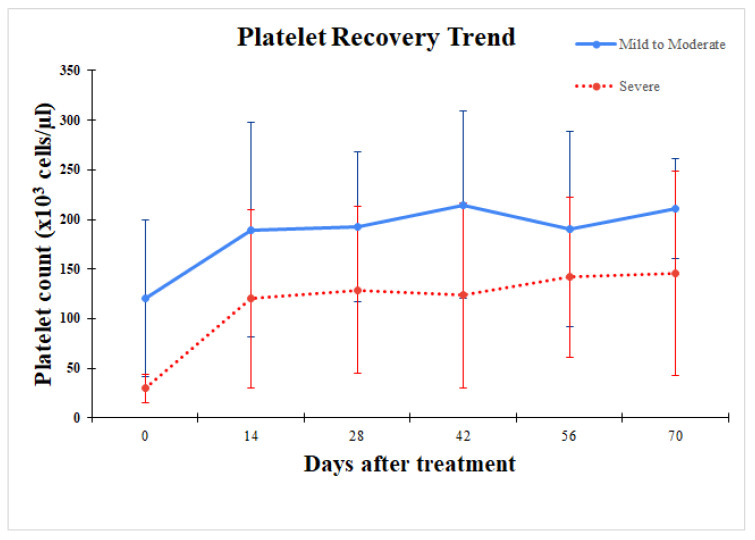
Trend of increased number of platelets before and after treatment in the mild-to-moderate thrombocytopenia group (solid line) compared to the severe thrombocytopenia group (dot line).

On day 0, the animals were divided into two groups based on their PLT to monitor recovery trends. The first group exhibited mild to moderate thrombocytopenia (platelet count > 50,000/µL, N = 17), while the second group exhibited severe thrombocytopenia (platelet count < 50,000/µL, N = 17) (Figure 2). The results demonstrated that both groups exhibited similar trends, where platelet levels increased significantly after 14 days of treatment and subsequently stabilized (Figure 2). In the mild to moderate group, the average PLT reached normal levels by day 14, whereas the severe group achieved normal levels by day 70.

**Figure 3:**
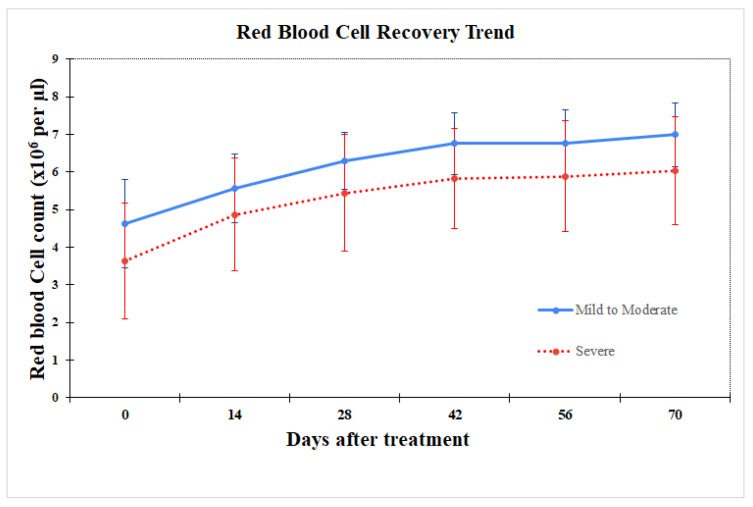
Trend of increased number of red blood cells before and after treatment in the mild-to-moderate group (solid line) compared to the severe group (dot line).

The trend of anemia recovery was comparable between mild to moderate and severe thrombocytopenia groups, as mentioned above. A dramatic improvement in RBC of both groups were observed between day 0 and day 28, followed by a gradual increase (Figure 3). In the mild to moderate group, the average RBC reached normal levels by day 14, whereas the severe group achieved normal levels of RBC by day 28 (Figure 3).

## 4. Discussion

### 4.1. Blood Pathogen Infections

The present study revealed a high prevalence of blood pathogen infection in almost half of the dog population, which is consistent with a previous study in the same area [2,3]. The majority of pathogens was *E. canis*, followed by *A. platys* and *B. vogali*, which is consistent with other reports throughout Thailand [2,3,7,24]. The incidence of multiple blood parasite infections in dogs has risen, particularly in tropical and subtropical areas, due to the brown dog tick (*Rhipicephalus sanguineus*), the common vector of transmission of these blood pathogens [2,3]. The brown dog tick also transmits *Hepatozoon canis*, which can lead to hepatozoonosis in dogs [3]. A report from Bangkok, Thailand revealed a higher prevalence of *H. canis* in stray cats (32.3%) than in stray dogs (11.4%) [25]. In the present study, the prevalence of *H. canis* in dogs was only 1.06%.

Mosquitoes widely distribute filariasis, a vector-borne zoonotic disease in tropical areas. Numerous filarial nematodes are known to cause various diseases. A recent study revealed the identification of filarial nematode species in mosquitoes near cattle farms in Nakhon Si Thammarat, Thailand [26], the same province as the dog shelter in the present study. There was no PCR detection of filarial nematodes in the sheltered dogs in the study, likely due to the regular routine of subcutaneous injection of ivermectin (400 mcg/kg). McCall and colleagues reported that dogs infected with *Dirofilaria immitis* were microfilaria count negative and had no rebound at least 21.3 months after receiving treatment with doxycycline 10 mg/kg/day for 30 days and ivermectin once a month [27].

Additionally, we detected DNA of *Trypanosome* spp. in the one dog blood sample. It has been noted that trypanosomes were rarely detected in dogs in endemic areas including India, Indonesia, and Malaysia [28] and a report from Germany in 2012 [29]. Conversely, trypanosomes in dogs have not been detected in Thailand, Philippines, and Vietnam for many years, with one report more than a decade ago [28]. The dog who tested positively for *E. canis* and *Trypanosome* spp. antigens in the present study did not show any significant clinical signs or blood profile changes (unpublished data). However, trypanosomes have recently been detected in other species, with around 2% in possible vectors (biting flies), 3% in ruminants, 6% in bats, and 18% in rats across Thailand [30,31,32,33].

### 4.2. Blood Profiles

For the blood profiles, the most typical hematological change observed in triple blood pathogen infection in this study was thrombocytopenia, which was found in more than 90% of cases. Regarding anemia, lowering of HCT and HGB were more frequently observed (88%) than a low RBC. Blood pathogen infections may have a dose-dependent relationship with anemia [3]. Thrombocytopenia and anemia are highly associated with these three pathogens, particularly *E. canis*, which seems to affect thrombocytes more than other pathogens, while *A. platys* is more specific to red blood cells [3]. Villaescusa et al. reported ehrlichiosis in dogs had high association with elevation of T-cells and cytotoxic T (Tc) cells, as well as the total amount of Tc cells in blood circulation [34]. In healthy dogs, the number of T helper cells, the total and relative counts of B lymphocytes were higher compared to ehrlichiosis dogs [34]. The research showed that dogs infected with blood parasites had reduced lymphocyte counts [34]; however, lymphocyte counts were not altered by multiple blood pathogen infections in the present study, while monocyte counts were reduced and eosinophil counts were increased.

Recently, consensus statements of the American College of Veterinary Internal Medicine (ACVIM) suggested immune thrombocytopenia (ITP) is a disorder which is commonly found in dogs but is rare in cats [35]. Severe thrombocytopenia can cause life-threatening hemorrhaging. Secondary ITP treatment aims to eliminate underlying causes firstly, while treatment for the associated immune-mediated disorder could be started using glucocorticoid with or without vincristine, human IV immunoglobulin, and blood product transfusion [35]. In the present study, on day 0, the average PLT was extremely low at 29,760/µL in the severe thrombocytopenia group. This could be mediated by providing treatment for the secondary ITP following the ACVIM guideline. The treatment goal of ITP is PLT count more than 100,000/µL without bleeding, whereas PLT less than 30,000/µL is considered as no response [35]. However, the average PLT in severe thrombocytopenia group in the present study was raised to 120,000/µL after treatment with doxycycline on day 14, then slowly increased to a normal level by day 70. In this study, the course of treatment began prior to the release of the consensus. The PLT could be increased to a normal level before day 70 if the ITP treatment is applied.

In the present study, only AST and ALP showed a significant reduction after treatment, suggesting an association with triple pathogen infection. ALP and AST are found in many tissues, with higher amounts of ALP in the liver, bile ducts, and bone, while AST is specific to the liver, heart, pancreas, and muscles. The increase in these two enzymes has also been observed in previous studies involving *E. canis* [36,37] and canine babesiosis [38].

However, when considering the prevalence of possible liver injury, this study found it to be less than 12%, which is lower than the 30–65% observed in a previous study [36]). In the present study, renal injury parameters such as elevated BUN were found in around 20% of cases, similar to a previous study, while elevated creatinine was found in only 8%, which is lower than the 33% reported in another study [36,39].

### 4.3. Treatment and Further Studies

Although doxycycline is not typically the first-choice medicine to treat canine babesiosis, it has been recently used effectively to treat human babesiosis (*B. venatorum*) [40]. Additionally, combining doxycycline with other drugs such as diminazine aceturate, metronidazole, and clindamycin has been shown to be more effective in treating canine babesiosis (*B. gibsoni*) compared to standard treatment (atovaquone and azithromycin) [41,42], as well as in co-infections with human babesiosis (*B. divergens*) and anaplasmosis (*A. phagocytophilum*) [43]. The present study showed that after 28 days of treatment, all three pathogens, *B. vogeli*, *E. canis*, and *A. platys*, were undetectable, and hematology and serum biochemistry parameters were significantly improved, suggesting that the dose of doxycycline used, i.e., 10 mg/kg/day once daily for 28 days, was sufficient to treat dogs with the triple blood pathogen infection.

Antimicrobial drugs have been widely used to treat many blood pathogens, though there is a concern about the development of drug-resistant pathogens. Recent studies have reported the presence of antimicrobial resistance genes and resistant blood pathogens, which could pose a threat to animal and human health [44,45,46]. Research funding for the present study was limited, particularly due to the expenses associated with sampling, diagnosing, and treating a large number of dogs. Future studies should focus on drug-resistant blood pathogens and alternative antimicrobial drugs, as current knowledge and reports of resistant pathogens are limited.

## 5. Conclusions

The present study suggests that triple blood pathogen infection with *B. vogeli*, *E. canis*, and *A. platys*, can cause severe anemia and thrombocytopenia in dogs, and treatment with 10 mg/kg/day doxycycline monotherapy, orally and once daily for 28 days, is effective and safe without combination with other treatments. In addition to improving the health and welfare of sheltered, stray, free-roaming, and pet dogs naturally infected with multiple blood pathogens, this study would have a favorable impact on management of the co-infections in endemic regions with limited veterinary resources.

## Figures and Tables

**Table 1:** Primers, primer concentration, and polymerase chain reaction program for blood parasite detection in dogs.

Pathogen	Gene	Primer	Sequences (5′ to 3′)	Product Size (bp)	Annealing Temperature
*Babesia vogeli*	18S rRNA	F	GTGAACCTTATCACTTAAAGG	~600	54 °C
		R	CAACTCCTCCACGCAATCG		
*Ehrlichia canis*	virB9 protein gene	F	CCATAAGCATAGCTGATAACCCTGTTACAA	380	54 °C
		R	TGGATAATAAAACCGTACTATGTATGCTAG		
*Hepatozoon canis*	18S rRNA	F	CCTGGCTATACATGAGCAAAATCTCAACTT	737	54 °C
		R	CCAACTGTCCCTATCAATCATTAAAGC		
*Anaplasma platys*	GroeL	F	TAGCTAAGGAAGCGTAGTCCGA	275	58 °C
		R	AATAGCCGCAGCGAGCGGTTCC		
*Trypanosoma* spp.	ITS1	F	CCGGAAGTTCACCGATATTG	250–700	58 °C
		R	TGCTGCGTTCTTCAACGAA		
Filarial nematodes	COI	F	TGATTGGTGGTTT TGGTAA	690	52 °C
		R	ATAAGTACGAGTATCAATATC		

**Table 2:** Prevalence of blood parasite infection.

	N	%
Negative	214	57.07%
Single infection	87	23.20%
*E. canis*	79	21.07%
*A. platys*	3	0.80%
*H. canis*	2	0.53%
*B. vogeli*	3	0.80%
*Trypanosome* spp.	0	0.00%
*Dirofilaria* spp.	0	0.00%
Double infection	40	10.67%
*E. canis* + *A. platys*	14	3.73%
*E. canis* + *B. vogeli*	23	6.13%
*E. canis* + *Trypanosome* spp.	1	0.27%
*E. canis* + *H. canis*	2	0.53%
Triple infection *A. platys* + *E. canis* + *B. vogeli*	34	9.07%
Total infection	161	42.93%

Abbreviations: *E. canis*: *Ehrlichia canis*, *A. platys*: *Anaplasma platys*, *H. canis*: *Hepatozoon canis*, *B. vogeli*: *Babesia canis vogeli*.

**Table 3:** Red blood cell and platelet parameters before and after doxycycline treatment, shown as mean ± SD.

Day	RBC ×10^6^/µL	HCT %	HGB g/dL	RET ×10^3^/µL	PLT ×10^3^ cell/µL	MPV fL
0	4.14 ± 1.44 ^a^	24.21 ± 7.84 ^a^	8.17 ± 3.21 ^a^	105.89 ± 125.42	75.35 ± 72.61 ^a^	16.09 ± 3.65 ^a^
14	5.15 ± 1.21 ^b^	29.83 ± 7.56 ^b^	10.33 ± 2.53 ^b^	72.87 ± 42.01	143.71 ± 101.36 ^b^	14.04 ± 2.33 ^b^
28	5.87 ± 1.28 ^c^	33.35 ± 7.08 ^c^	11.64 ± 2.69 ^c^	64.70 ± 37.23	162.24 ± 85.74 ^b^	13.73 ± 1.87 ^b^
42	6.28 ± 1.19 ^c,d^	35.41 ± 7.68 ^d^	12.45 ± 2.71 ^d^	54.05 ± 29.62	171.65 ± 101.21 ^b,c^	13.57 ± 1.68 ^b^
56	6.35 ± 1.27 ^d^	35.76 ± 7.91 ^d^	12.57 ± 2.77 ^d^	56.29 ± 28.12	171.32 ± 73.81 ^b,c^	13.73 ± 2.04 ^b^
70	6.49 ± 1.25 ^d^	36.74 ± 7.97 ^d^	14.22 ± 8.36 ^d^	66.63 ± 51.54	180.68 ± 106.28 ^c^	13.62 ± 1.61 ^b^
*p*-value	<0.01	<0.05	<0.05	>0.05	<0.01	<0.01
Reference value	5–8	35–61	12–20	10–110	125–400	8–13

^a,b,c,d^ represents statically significant difference (*p* < 0.05). Abbreviations: RBC = red blood cell count, HCT = hematocrit, HGT = hemoglobin concentration, RET = reticulocyte count, MPV = mean platelet volume.

**Table 4:** Number and percentage of the triple pathogen infected dogs (N = 34) recovered from anemia and thrombocytopenia after the doxycycline treatment.

	Anemia			Thrombocytopenia
Day	RBC < 5 × 10^6^/µL	HCT < 35%	HGB < 12 g/dL	Platelet < 125 × 10^3^/µL
0	23 (67.65%) ^a^	30 (88.24%) ^a^	30 (88.24%) ^a^	31 (91.18%) ^a^
14	15 (44.12%) ^b^	23 (67.65%) ^b^	24 (70.89%) ^b^	16 (47.06%) ^b^
28	9 (26.47%) ^b,c^	16 (47.06%) ^c^	16 (47.06%) ^c^	13 (38.24%) ^b^
42	7 (20.59%) ^c^	14 (41.18%) ^c^	13 (38.24%) ^c^	12 (35.29%) ^b^
56	7 (20.59%) ^c^	13 (38.24%) ^c^	12 (35.29%) ^c^	11 (32.35%) ^b^
70	4 (11.76%) ^c^	12 (35.29%) ^c^	12 (35.29%) ^c^	11 (32.35%) ^b^
*p*-value	<0.02	<0.02	<0.02	<0.01

^a,b,c^ represents statically significant difference (*p* < 0.05). *p*-value < 0.01 represent very significant difference. Abbreviations: RBC = red blood cell count, HCT = hematocrit, HGB = hemoglobin concentration.

**Table 5:** White blood cells parameters before and after the doxycycline treatment, shown as mean ± SD.

Day	WBC ×10^3^/µL	NEU ×10^3^/µL	LYM ×10^3^/µL	MON ×10^3^/µL	EOS ×10^3^/µL	BAS ×10^3^/µL
0	12.95 ± 6.05	8.40 ± 4.88	2.76 ± 1.78	1.23 ± 0.71 ^a^	0.49 ± 0.43 ^a^	0.07 ± 0.008
14	11.04 ± 4.56	6.72 ± 3.55	2.74 ± 1.85	0.84 ± 0.46 ^b^	0.64 ± 0.45 ^a^	0.11 ± 0.10
28	11.53 ± 3.89	6.73 ± 3.13	2.42 ± 1.31	0.76 ± 2.63 ^c^	0.87 ± 0.65 ^a^	0.63 ± 2.70
42	11.27 ± 3.74	6.98 ± 2.53	2.32 ± 1.09	0.75 ± 0.37 ^d^	1.07 ± 0.66 ^b^	0.15 ± 0.18
56	11.95 ± 4.10	7.31 ± 2.57	2.48 ± 1.18	0.76 ± 0.38 ^d^	1.29 ± 1.21 ^b^	0.12 ± 0.13
70	11.93 ± 3.91	7.64 ± 3.68	2.63 ± 1.24	0.72 ± 0.38 ^d^	1.42 ± 1.12 ^b^	0.09 ± 0.09
*p*-value	>0.05	>0.05	>0.05	<0.01	<0.01	>0.05
Reference value	5–16	2–11	1–5	0.1–1	0.06–1	0–0.1

^a,b,c,d^ represents statically significant difference (*p* < 0.05). Abbreviations: MON = monocytes, EOS = eosinophils, WBC = total white blood cells, NEU = neutrophils, LYM = lymphocytes, BAS = basophils.

**Table 6:** Serum biochemistry of the dogs (N = 34) before and after treatment, shown as mean ± SD.

Day	BUN mg/dL	CRE mg/dL	AST U/L	ALT U/L	ALP U/L	TP g/dL
0	20.29 ± 19.01	1.03 ± 0.67	34.44 ± 12.69 ^a^	36.03 ± 29.34	66.35 ± 54.07 ^a^	10.13 ± 14.32
14	17.08 ± 13.73	0.97 ± 0.60	25.06 ± 7.21 ^b^	30.68 ± 20.12	68.09 ± 52.57 ^a^	9.04 ± 11.76
28	14.40 ± 5.69	1.03 ± 0.53	24.47 ± 7.16 ^b^	30.97 ± 23.13	50.88 ± 27.11 ^a,b^	6.61 ± 1.25
42	16.02 ± 5.26	1.04 ± 0.40	26.26 ± 7.74 ^b^	33.38 ± 31.71	42.56 ± 19.04 ^a,b^	7.77 ± 8.78
56	16.27 ± 6.02	1.07 ± 0.42	25.39 ± 7.97 ^b^	40.88 ± 40.78	39.61 ± 17.97 ^a,b^	6.10 ± 1.13
70	18.72 ± 4.98	1.09 ± 0.49	29.24 ± 10.69 ^b^	40.15 ± 35.65	37.24 ± 21.07 ^b^	6.43 ± 1.19
*p*-value	>0.05	>0.05	<0.01	>0.05	<0.05	>0.05
Reference value	7–26	0.5–1.8	0–56	10–95	23–150	5–7

^a,b^ represents statically significant difference (*p* < 0.05). Abbreviations: AST = aspartate transaminase, ALP = alkaline phosphatase, BUN = blood urea nitrogen, CRE = creatinine, ALT = alanine aminotransferase, TP = total protein.

**Table 7:** Number and percentage of the dogs with hepatic and/or renal injuries before and after the doxycycline treatment.

	Hepatic Injury			Renal Injury	
Day	ALT > 95 U/L	AST > 56 U/L	ALP > 150 U/L	BUN > 26 mg/dL	CRE > 1.8 mg/dL
0	1 (2.94%)	2 (5.88%)	4 (11.76%)	7 (20.59%)	3 (8.82%)
14	1 (2.94%)	0 (0.00%)	3 (8.82%)	3 (8.82%)	2 (5.88%)
28	1 (2.94%)	0 (0.00%)	0 (0.00%)	1 (2.94%)	2 (5.88%)
42	1 (2.94%)	0 (0.00%)	0 (0.00%)	0 (0.00%)	1 (2.94%)
56	2 (5.88%)	0 (0.00%)	0 (0.00%)	0 (0.00%)	1 (2.94%)
70	2 (5.88%)	1 (2.94%)	0 (0.00%)	0 (0.00%)	1 (2.94%)
*p*-value	>0.05	>0.05	>0.05	>0.05	>0.05

No significant difference, *p* > 0.05. Abbreviations: AST = aspartate transaminase, ALP = alkaline phosphatase, BUN = blood urea nitrogen, CRE = creatinine, ALT = alanine aminotransferase.

## Data Availability

No new data were created or analyzed in this study. Data sharing is not applicable to this article.
